# Subthreshold micropulse laser photocoagulation versus half-dose photodynamic therapy for acute central serous chorioretinopathy

**DOI:** 10.1186/s12886-022-02331-z

**Published:** 2022-03-08

**Authors:** Miao Zeng, Xiao Chen, Yanping Song, Chunyan Cai

**Affiliations:** 1 Department of Ophthalmology, General Hospital of Central Theater Command of PLA (Clinical Medicine Center of Eye Fundus Laser in Hubei Provience), Wuhan, Hubei China; 2 Aier Eye Hospital of Wuhan University, 481 Zhongshan Road, Wuhan, 430070 Hubei China

**Keywords:** Central serous chorioretinopathy, Subthreshold micropulse, Laser photocoagulation, Photodynamic therapy

## Abstract

**Purpose:**

To compare the efficacy and safety of subthreshold micropulse (STMP) laser photocoagulation and half-dose photodynamic therapy (PDT) in the treatment of acute central serous chorioretinopathy (CSC).

**Methods:**

A total of 39 acute CSC patients were included. 18 patients were treated with STMP laser and 21 patients were treated with half-dose PDT. The main outcome measures were best-corrected visual acuity (BCVA) according to Early Treatment Diabetic Retinopathy Study (ETDRS) chart, the proportion of eyes with complete resolution of subretinal fluid, the number of treatments, and the retinal sensitivity by microperimetry during the 12-month study period.

**Results:**

The mean number of treatments during the 12-month period was 1.6 in STMP group and 1.3 in half-dose PDT group, respectively (*P* = 0.174). The proportion of eyes with complete resolution of subretinal fluid was 83.3% in STMP group compared with 90.5% in half-dose PDT group at 12-month (*P* = 0.647). The mean BCVA at 12-month after treatment was 75.28 ± 12.58 for STMP group and 76.62 ± 11.57 for half-dose PDT group, respectively (*P* = 0.731). No statistically significant difference was found in the mean retinal sensitivity between the two groups during the 12 months follow-up (*P* = 0.701 at 3 months; *P* = 0.725 at 6 months; *P* = 0.695 at 12 months).

**Conclusions:**

Subthreshold micropulse Laser Photocoagulation is as effective as half-dose PDT for acute CSC, while minimizing the damage effect on retinal.

## Introduction

Central serous chorioretinopathy (CSC) is a common ocular disease characterized by a serous neurosensory retinal detachment in the posterior pole, often accompanied with retinal pigment epithelial (RPE) detachment [1]. Currently, the pathogenesis of CSC is still unclear [2]. Benefit from the widespread use of indocyanine green angiography (ICGA) in ocular fundus disease, we can further understand the pathogenesis of CSC. Base on ICGA findings, several studies demonstrate the evidences of choroidal circulation abnormalities in addition to RPE in CSC, leading to multifocal areas of choroidal vascular hyperpermeability [3–8]. Patients with CSC usually experience some visual symptoms such as visual acuity decreased, metamorphopsia, micropsia, dyschromatopsia and relative central scotoma [8]. Most cases of acute CSC are self-limiting with spontaneous resolution of subretinal fluid within 3–4 months [9]. However, up to 30–50% of patients, symptoms of serous retinal detachment may remain or recur within the first year [10]. Furthermore, a few of patients may suffer visual loss because the development of RPE atrophy or choroidal neovascularizations (CNV) [11].

The most usually performed treatments for CSC are photodynamic therapy (PDT) with verteporfin and subthreshold micropulse laser (STMP) treatment. Nevertheless, PDT may increase the risk of RPE atrophy, choroidal ischemia, and secondary CNV [12–14]. These adverse effects may restrict the use of PDT for acute CSC. In the past few years, several studies had reported that half-dose photodynamic therapy (PDT) with verteporfin were effective for the treatment of acute CSC by reducing subretinal fluid and increasing visual acuity in most patients, while minimizing side effects [9, 15], however, it was expensive and unavailable in some regions.

Subthreshold micropulse (STMP) is a laser emission technique which is made up of trains of repetitive ultrashort laser pulses. It creates sublethal cellular thermal effect at the RPE without heat conduction to surrounding retinal tissue [16], and therefore it diminishes the risk of iatrogenic thermal damage. STMP laser therapy for acute CSC has been previously described [17, 18]. These studies have reported that STMP laser is effective for actue CSC, while few side effects occurred. Compared with PDT, the costs of STMP laser therapy are much lower for patients. However, until now there are few studies focused on comparing the results of STMP laser treatment and PDT treatment for aucte CSC.

This study attempted to evaluate the effect of STMP laser and half-dose photodynamic therapy (PDT) for acute CSC, and to analyze whether there are differences in efficacy between STMP laser and PDT in the treatment of acute CSC.

## Materials and methods

This study was a retrospective, nonrandomized, controlled clinical study on 39 patients with acute CSC. It was conducted at the Department of Ophthalmology, General Hospital of Central Theater Command of PLA. This study was approved by the Institutional Review Boards of General Hospital of Central Theater Command of PLA. The research adhered to the tenets of Declaration of Helsinki. Informed consents were obtained from all subjects, when informed about the risk of complications. Patients were divided into two groups according to the treatment.

All patients with acute CSC were enrolled from June 2017 to April 2019. Inclusion criteria included: (1) patient age ≥ 18 years; (2) the symptoms of CSC for 3 months or less; (3) presence of subretinal fluid involving the fovea, with or without RPE detachment on optical coherence tomography (OCT); (4) presence of active angiographic leakage in fundus fluorescein angiography(FFA) caused by CSC. The following were exclusion criteria: (1) any evidence of choroidal neovascularization on FFA and ICGA; (2) any other associated ocular and macular disease influencing visual acuity; (3) history of any intraocular surgery; (4) received previous treatments including PDT, focal thermal laser photocoagulation, intravitreal injection of steroid or anti- VEGF agent.

The baseline ophthalmologic examination was performed in each patient, including best-corrected visual acuity (BCVA) by the standard Early Treatment Diabetic Retinopathy Study (ETDRS) chart, slit-lamp examination, indirect fundoscopy, color fundus photography (TRC-NW7SF; Topcon, Japan), OCT (3D OCT-2000; Topcon, Japan), microperimetry (MP-1; Nidek Italy), FFA and ICGA (Heidelberg Engineering, Heidelberg, Germany). Diagnosis of acute CSC was based on findings on indirect ophthalmoscopy, FFA, ICGA, and OCT. Central foveal thickness (CFT) was defined as the distance between the inner neurosensory retinal surface and the inner border of the RPE at the central foveal. Retinal sensitivity was performed using microperimetry within 12 degrees of the retinal central in a dark room. The mean retinal sensitivity was evaluated.

PDT was performed using the half-dose of verteporfin (Visudyne; Novartis, Basel, Switzerland), which is 3 mg/m^2^ verteporfin. Verteporfin was infused over 10 min, followed by laser delivery at 15 min from the start of infusion. The laser light at 689 nm was delivered to the area of choroidal hyperperfusion as observed in ICGA for 83 s with an energy of 50 J/cm^2^. The laser spot size was set at a smallest size of covering the whole area of choroidal hyperperfusion. After treatment, the patients were instructed to avoid strong light for 3 days.

STMP photocoagulation treatment was performed with an 810-nm diode laser (OcuLight SLx; Iridex Corp, USA), and laser light was delivered via a slit-lamp adapter though an Area Centralis Laser contact lens (Volk Optical Inc, Mentor, USA). The laser power was determined in each patient by creating a threshold burn with the lowest energy required to make a visible “test burn” at the nasal mid periphery of retina. Then the laser was applied at a lower energy level and changed to the micropulse mode. The laser apparatus were: 125um spot, 0.2-s exposure duration, 15% duty cycle. About 100 pulse envelope were applied at each leakage site identified by FFA [16].

Repeated treatment was performed at least 3 months later from baseline if the subretinal fluid of CSC was not or incompletely resolved 3 months after treatments. During follow-up, patients were not allowed to change groups.

Follow-up examinations with BCVA, slit-lamp, indirect fundoscopy, OCT were performed at baseline and each monthly visit. FFA, ICGA, and microperimetry were performed at baseline and control visit at month 3, 6, 9, and 12.

The primary outcome measure of the study was the proportion of eyes with complete absorption of the macular subretinal fluid (SRF) at 12 months. Secondary outcome measures were the numbers of treatments and the serial changes in BCVA, CFT, and retinal sensitivity.

Statistical analysis was performed using SPSS 13.0. Comparisons of mean BCVA, CFT and central retinal sensitivity before and after treatment within each group were conducted using the repeated measure ANOVA. All the numerical variables were tested for normality. The student *t*-test or Mann–Whitney *U* test was used for comparison of the numerical variables between the two groups. The Mann–Whitney *U* test was used for comparison of the ordinal variables between the two groups. The Fisher exact test was used to compare the categorical variables. A *P* value of < 0.05 was considered to be statistically significant.

## Results

The average age was 43.4 ± 9.8 years. 27 patients were male (69.2%), and 12 patients were female (30.8%). The mean follow-up period was 16.4 ± 3.8 months.18 eyes were included in the STMP group, 21 eyes were included in the half-dose PDT group. No patients were lost to follow-up in the study. Baseline characteristics of the two groups are detailed in Table 1.

**Table 1:** Baseline characteristics and demographics of patients with acute CSC

	STMP Group	Half-dose PDT Group	*P* value
Variables	(*n* = 18)	(*n* = 21)	
Age, mean ± SD, (years)	37.22 ± 7.83	38.76 ± 8.08	0.582
Gender, (n)			
Male	13	14	
Female	5	7	0.742
Duration of symptoms,			
mean ± SD, (days)	25.06 ± 16.09	21.33 ± 15.63	0.359
Baseline BCVA, mean ± SD,			
(ETDRS)	63.67 ± 9.79	62.81 ± 8.52	0.772
Baseline CFT, mean ± SD,			
(μm)	427.28 ± 52.23	423.38 ± 47.39	0.808
Baseline retinal			
sensitivity, mean ± SD, (dB)	12.29 ± 1.88	12.61 ± 1.95	0.601
Presence of macular PED, (n)	3(16.7%)	5(23.8%)	0.702
Location of leakage site, (n)			
Subfoveal	2	4	
Juxtafoveal	14	15	
Extrafoveal	2	2	0.789
Number of leakage point, (n)			
1	15	16	
2	3	4	
3	0	1	0.622

*CSC* central serous chorioretinopathy, *STMP* subthreshold micropulse, *PDT* photodynamic therapy, *SD* standard deviation, *BCVA* best-corrected visual acuity, *ETDRS* Early Treatment Diabetic Retinopathy Study, *CFT* central foveal thickness, *PED* pigment epithelial detachment

In the STMP group, the resorption of the macular SRF was complete in 11 eyes (61.1%) at 1 month, 15 eyes (83.3%) at 3 months, 12 eyes (66.7%) at 6 months, 16 eyes (88.89%) at 9 months, and 15 eyes (83.3%) at 12 months. During the 12-month follow-up period, the macular SRF had recurred in three patients, and persisted in two patients. The mean times of STMP laser were 1.6 times. In the half-dose PDT group, complete absorption of the macular SRF was achieved in 17 eyes (81.0%) at 1 month, 20 eyes (95.2%) at 3 months, 19 eyes (90.5%) at 6 months, 18 eyes (85.71%) at 9 months, and 19 eyes (90.5%) at 12 months. The macular SRF had recurred in two patients, and persisted in one patient during the 12-month period. The mean times of half-dose PDT were 1.3 times. The proportion of eyes with complete absorption of the macular SRF and the number of treatments between the two groups were no significant difference (*P* = 0.647, Fisher exact test; *P* = 0.174, Mann–Whitney U test, respectively).

**Fig. 1:**
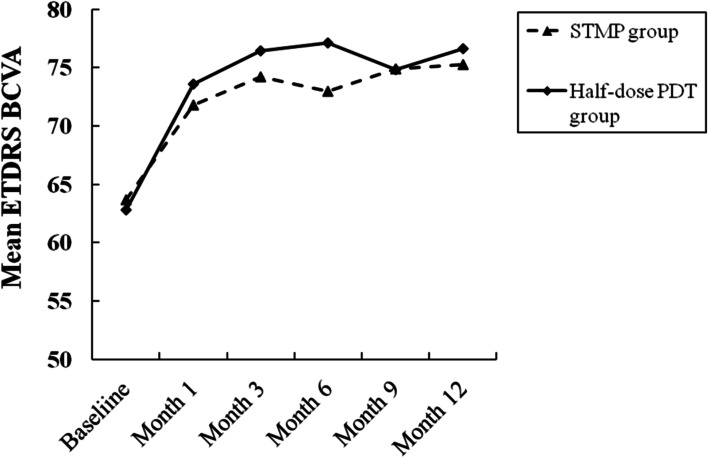
Graph showing the serial changes in mean Early Treatment Diabetic Retinopathy Study(ETDRS) best-corrected visual acuity(BCVA) of patients in the STMP group (dashed line) and in the half-dose PDT group (solid line)

In the STMP groups, the mean ETDRS BCVA was 63.67 ± 9.79 at baseline, and improved to 71.78 ± 12.23 at 1 month, 74.22 ± 11.66 at 3 months, 73.00 ± 13.73 at 6 months, 74.89 ± 12.42 at 9 months, 75.28 ± 12.58 at 12 months, respectively. The improvement of mean BCVA was significant over the 12-month period (*F* = 2.311 *P* = 0.049, ANOVA). Multiple comparison analysis indicated that the mean BCVA improved significantly during follow-up compared with the baseline values (*P* = 0.047 at 1 month; *P* = 0.01 at 3 months; *P* = 0.023 at 6 months; *P* = 0.007 at 9 months; *P* = 0.005 at 12 months, ANOVA). At 12 months, the ETDRS BCVA was improved by ≥ 5 letters in 15 eyes, improved by ≥ 15 letters in 6 eyes, remained unchanged (increased or decreased less than 5 letters) in 3 eyes, none of eyes worsened ≥ 5 letters (Table 2). In the half-dose PDT group, the BCVA improved significantly over the 12-month period (*F* = 5.696 *P* < 0.001, ANOVA). The mean BCVA was 62.81 ± 8.52 at baseline, 73.62 ± 10.10 at 1 month, 76.43 ± 10.46 at 3 months, 77.10 ± 10.79 at 6 months, 74.86 ± 10.83 at 9 months, 76.62 ± 11.57 at 12 months. The mean BCVA improved significantly during follow-up compared with the baseline values (*P* = 0.001 at 1 month; *P* < 0.001 at 3 months; *P* < 0.001 at 6 months; *P* < 0.001 at 9 months; *P* < 0.001 at 12 months, ANOVA). At 12 months, the BCVA increased at least 5 letters in 19 eyes, improved by ≥ 15 letters in 8 eyes, unchanged in 2 eyes, and none of eyes decreased ≥ 5 letters (Table 2). There was no significant statistical difference in the mean BCVA between the two groups during follow-up (*P* = 0.610 at 1 month; *P* = 0.537 at 3 months; *P* = 0.304 at 6 months; *P* = 0.993 at 9 months, *P* = 0.731 at 12 months, *t*-test). The changes in BCVA over the 12-month study period are shown in Fig. 1.

**Table 2:** Proportion of complete absorption of macular SRF, change in BCVA, and number of treatment over the 12-month period both the STMP group and the half-dose PDT group

STMP Group	Half-dose PDT Group	*P* value	
Variables	(*n* = 18)	(*n* = 21)	
Complete absorption of			
macular SRF at month 12,			
(n)	15(83.3%)	19(90.5%)	0.647
Change in BCVA at month 12,			
(n), (ETDRS)			
Gained ≥ 15 letters	6 (33.3%)	8(38.1%)	0.757
Gained ≥ 5 letters	15(83.3%)	19(90.5%)	
No change	3(16.7%)	2(9.5%)	0.506
Lost ≥ 5 letters	0	0	
Number of treatment over			
the 12-month period, (n)			
1	11	17	
2	5	3	
3	0	0	
4	2	1	0.174

*SRF* subretinal fluid, *BCVA* best-corrected visual acuity, *STMP* subthreshold micropulse, *PDT* photodynamic therapy, *ETDRS* Early Treatment Diabetic Retinopathy Study

**Fig. 2:**
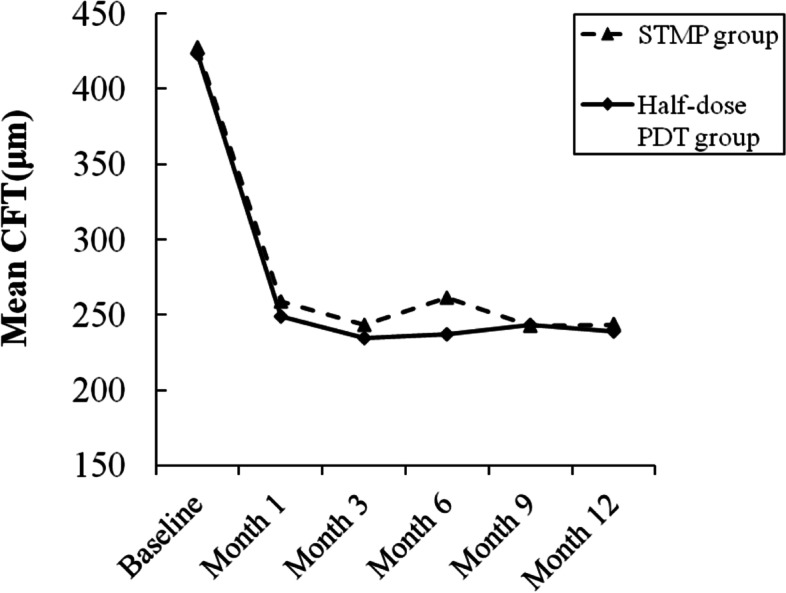
Graph showing the serial changes in mean central foveal thickness (CFT) of patients in the STMP group (dashed line) and in the half-dose PDT group (solid line)

The mean CFT in the STMP group was 427.28 ± 52.23 μm at baseline, and decreased to 259.06 ± 50.46 μm at 1 month, 243.17 ± 30.95 μm at 3 months, 261.56 ± 49.13 μm at 6 months, 242.72 ± 33.27 μm at 9 months, 243.67 ± 39.46 μm at 12 months (Fig. 2). The mean CFT was decreased significantly over the 12-month period (*F* = 50.680 *P* < 0.001, ANOVA). The CFT decreased significantly during follow-up compared with the baseline values (*P* < 0.001, ANOVA). In the half-dose group, the mean CFT was 423.38 ± 47.39 μm at baseline, 249.10 ± 52.36 μm at 1 month, 234.86 ± 31.31 μm at 3 months, 237.05 ± 41.10 μm at 6 months, 243.62 ± 43.89 μm at 9 months, 238.95 ± 34.12 μm at 12 months (Fig. 2). The mean CFT was decreased significantly over the 12-month period ( *F* = 65.503 *P* < 0.001, ANOVA). The CFT decreased significantly during follow-up compared with the baseline values (*P* < 0.001, ANOVA). There was no statistically significant difference in the mean CFT reduction between the two groups during follow-up (*P* = 0.551 at 1 month; *P* = 0.412 at 3 months; *P* = 0.098 at 6 months; *P* = 0.944 at 9 months; *P* = 0.691 at 12 months, *t*-test).

**Fig. 3:**
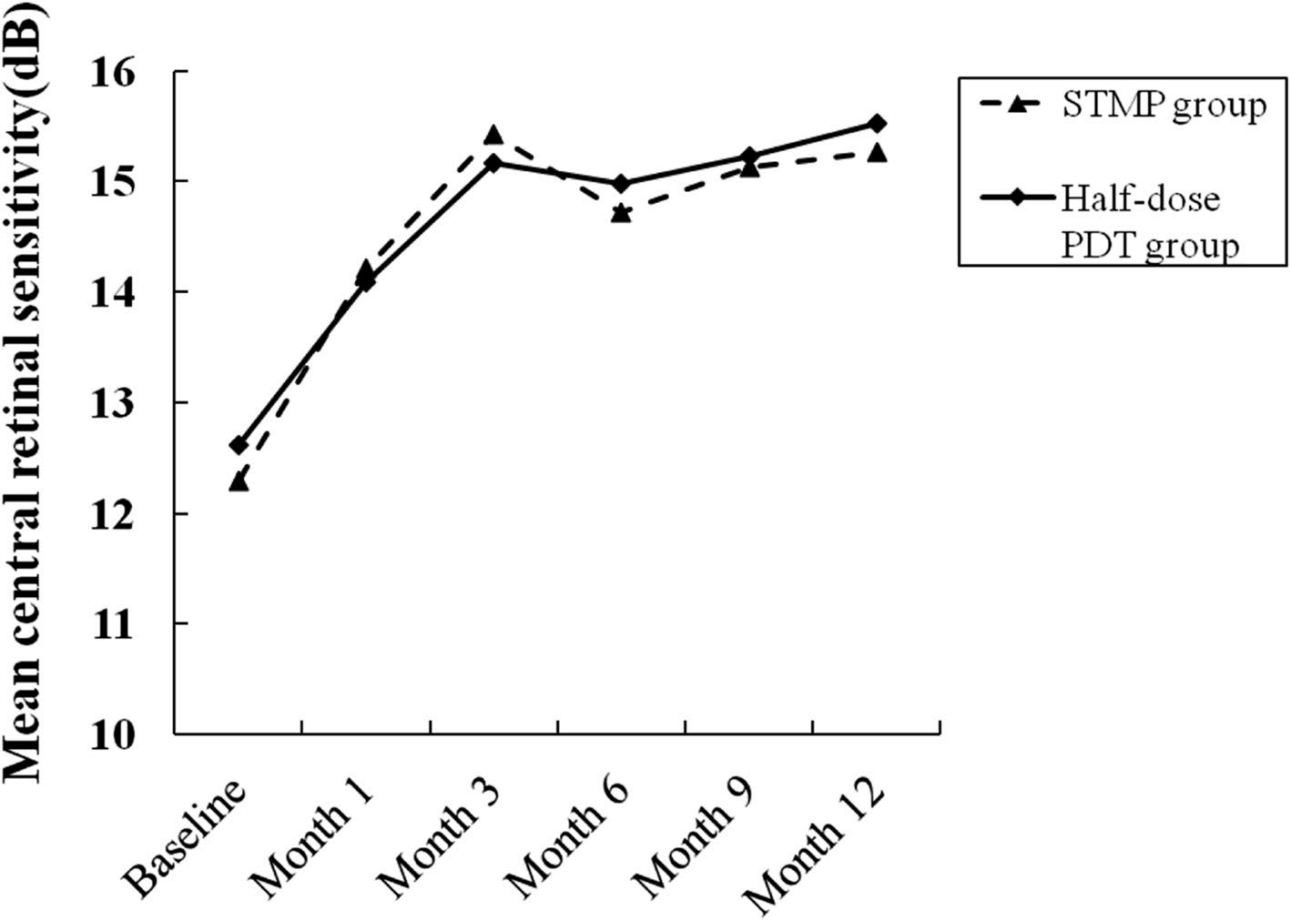
Graph showing the serial changes in mean central retinal sensitivity of patients in the STMP group (dashed line) and in the half-dose PDT group (solid line)

The mean retinal sensitivity in the STMP group was significantly improved during the 12-month period (*F* = 5.042 *P* < 0.001, ANOVA). It was 12.29 ± 1.88 dB at baseline, and improved to 14.21 ± 2.19 dB at 1 months (*P* = 0.011, ANOVA), 15.43 ± 2.16 dB at 3 months (*P* < 0.001, ANOVA), 14.72 ± 2.57 dB at 6 months (*P* = 0.001, ANOVA), 15.13 ± 2.23 dB at 9 months (*P* < 0.001, ANOVA), 15.26 ± 2.22 dB at 12 months (*P* < 0.001, ANOVA) (Fig. 3). In the half-dose group, the mean retinal sensitivity was improved during the study period (*F* = 6.254 *P* < 0.001, ANOVA). It was 12.61 ± 1.95 dB at baseline, and improved to 14.09 ± 1.80 dB at 1 months (*P* = 0.018, ANOVA), 15.17 ± 1.97 dB at 3 months (*P* < 0.001, ANOVA), 14.98 ± 2.09 dB at 6 months (*P* < 0.001, ANOVA), 15.23 ± 2.15 dB at 9 months (*P* < 0.001, ANOVA), 15.53 ± 2.01 dB at 12 months (*P* < 0.001, ANOVA) (Fig. 3). There was no statistical difference in retinal sensitivity between the two groups (*P* = 0.850 at 1 months; *P* = 0.701 at 3 months; *P* = 0.725 at 6 months; *P* = 0.887 at 9 months; *P* = 0.695 at 12 months, *t*-test).

No systemic or ocular side effects associated with treatment were observed in any of the cases during the follow-up period.

## Discussion

STMP laser is designed to target the RPE cells while minimizing the negative thermal effects on the neural retina and deeper structures [19]. STMP laser treated for CSC was first reported by Bandello et al. in 2003 (Invest Ophthalmol Vis Sci ARVO E-Abstract, 2003). The mechanisms of action of STMP laser in CSC is not clearly understood. It seems that STMP laser induces the production of intracellular biological factors that stimulate PRE function and speeds up absorption of the SRF which in turn leads to less photoreceptor damage [20]. Although several studies have confirmed the efficacy and safety of half-dose PDT and STMP laser in treating acute CSC [17, 18, 21, 22]. the clinical results compared between the two therapeutic methods have not been reported. In this retrospective study, we evaluate the changes in SRF, CFT, BCVA and central retinal sensitivity of acute CSC after treated by these two modalities respectively. In our study, we found that the anatomic success rates were slightly higher in half-dose PDT group compared with STMP group at 12-month, but there was no statistically significant difference between the two groups. However, at 1 month, the rate of resorption of SRF was significantly higher in half-dose PDT group (81.0%) than in STMP group (61.1%). The better anatomic outcomes after half-dose PDT treatment at short-term may be attributed to the fact that PDT targets the choroidal tissue, in contrast, STMP laser is thought to primarily produce an effect on the RPE. Chan et al. reported that the proportion was 79.5% at 1 month in acute CSC after treated by half-dose PDT [9], there results were consistent with our study. Kross et al. showed that the proportion of eyes with complete resolution of SRF was 37.5% at 6 weeks in CSC after treated by STMP laser [23]. the proportion was significantly lower than our results. We speculate that the different results may be due to the duration of CSC patients in their study was longer than ours. Our study showed that the proportion of eyes with recurrence of SRF was no significant difference in STMP group compared with half-dose PDT group during the 12-month follow-up period. Three eyes (16.7%) had recurred in STMP group versus 2 eyes (9.5%) in half-dose group, the proportion of recurrence was significantly higher in STMP group than half-dose PDT group. It could be due to the larger extent of PDT taking effect on choroid than STMP laser. In CSC, more extensive choroidal lesions could be exist, not limited to the area of leakage on FA [24]. The proportion of recurrence was higher compared with the results of other studies [9, 15, 18], however, the reason of the difference was unknown. Recurrence of SRF was occurred mainly between the 3-month and 9-month follow-up visits in most of those eyes. Until now, it remains controversial that STMP laser has the benefit of reducing recurrence of CSC. Some researchers demonstrated STMP laser could reduce the recurrence rate of eyes with CSC [16, 18], however, some other researchers deemed that STMP laser could not prevent the recurrence of CSC [25].

In this study, we found the mean BCVA was significantly improved compared with baseline in both groups at 12-month visit, and there were no statistical differences in the increase of letters between the two groups. Similar findings were observed in other studies on acute CSC patients who had received the treatments by half-dose PDT or STMP laser [9, 15, 17, 18]. However, some studies show a better result in visual acuity after half-dose PDT and STMP at the last follow-up compared with our study [15, 17, 18, 21]. It could be due to the worse visual acuity in our study at baseline. In this study, the improvement in BCVA fluctuated with a little drop at months 6 in STMP group and at months 9 in half-dose PDT group, which was accompanied by a small increase in CFT. It might be the result of recurrence of macular SRF at that time. A significant reduction of mean CFT was observed at each month after treatment in both groups. No significant difference in the mean CFT was found between the two groups. Compared with other studies [26], we found the CFT decreased faster in our patients treated with STMP laser. The reason for difference in speed of CFT recovery was unclear.

Compared with visual acuity, the retinal sensitivity is a more reliable index in evaluation of the function of more extensive macular area, including juxtafoeal and extrafoeal. Kim et al. demonstrated that the mean retinal sensitivity improved significantly after treated by half-dose PDT in acute CSC patients [15]. Fujita et al. performed half-dose PDT in 16 eyes with CSC and found that the mean retinal sensitivities at month 1 and month 3 after treatment had a great improvements compared with pretreatment [27]. Currently, there were few researches reported the changes in retinal sensitivity after treated by STMP laser in patients with acute CSC. Our study showed the mean retinal sensitivity improved significantly at each visit after treatment compared with baseline in both groups, and the retinal sensitivity was slight better in half-PDT group than STMP group at final visit, however, there was no statistical difference between the two groups. We speculated that the slight difference in retinal sensitivity was due to the higher absorption rate of SRF at early time after treatment in half-dose PDT group, which led to less photoreceptor damage.

The limitations of this study include the retrospective and nonrandomized design, the small sample size, a short follow-up period and the lack of an untreated control group for the assessment of the effect of each therapeutic method. Furthermore, some patients of acute CSC have a natural good prognosis, which might have created a bias in terms of anatomical outcomes. A randomized, placebo-controlled, long-term, large sample size clinical trial is necessary to ascertain the efficacy and safety of both STMP laser and half-dose PDT in acute CSC.

In conclusion, STMP laser and half-dose PDT can accelerate the absorption of SRF, improve visual acuity and central retinal sensitivity in patients with acute CSC without causing obvious retinal damage. Our study shows that STMP laser is a low-cost, effective and safe treatment for patients with acute CSC.

## Data Availability

The datasets used and/or analyzed during the current study are available from the corresponding author on reasonable request.

## References

[1] Spaide RF, Campeas L, Haas A, Yannuzzi LA, Fisher YL, Guyer DR, Slakter JS, Sorenson JA, Orlock DA (1996). Central serous chorioretinopathy in younger and older adults. Ophthalmology.

[2] Daruich A, Matet A, Dirani A, Bousquet E, Zhao M, Farman N, Jaisser F, Behar-Cohen F (2015). Central serous chorioretinopathy: Recent findings and new physiopathology hypothesis. Prog Retin Eye Res.

[3] Gemenetzi M, De Salvo G, Lotery AJ (2010). Central serous chorioretinopathy: an update on pathogenesis and treatment. Eye.

[4] Piccolino FC, Borgia L (1994). Central serous chorioretinopathy and indocyanine green angiography. Retina.

[5] Iida T, Kishi S, Hagimura N, Shimizu K (1999). Persistent and bilateral choroidal vascular abnormalities in central serous chorioretinopathy. Retina.

[6] Scheider A, Nasemann JE, Lund OE (1993). Fluorescein and indocyanine green angiographies of central serous choroidopathy by scanning laser ophthalmoscopy. Am J Ophthalmol.

[7] Prunte C, Flammer J (1996). Choroidal capillary and venous congestion in central serous chorioretinopathy. Am J Ophthalmol.

[8] Ross A, Ross AH, Mohamed Q (2011). Review and update of central serous chorioretinopathy. Curr Opin Ophthalmol.

[9] Chan WM, Lai TY, Lai RY, Liu DT, Lam DS (2008). Half-dose verteporfin photodynamic therapy for acute central serous chorioretinopathy: one-year results of a randomized controlled trial. Ophthalmology.

[10] Matet A, Daruich A, Zola M, Behar-Cohen F (2018). Risk Factors for Recurrences of Central Serous Chorioretinopathy. Retina.

[11] Shiragami C, Takasago Y, Osaka R, Kobayashi M, Ono A, Yamashita A, Hirooka K (2018). Clinical Features of Central Serous Chorioretinopathy With Type 1 Choroidal Neovascularization. Am J Ophthalmol.

[12] CardilloPiccolino F, Eandi CM, Ventre L (2003). Rigault de la Longrais RC, Grignolo FM: **Photodynamic therapy for chronic central serous ****chorioretinopathy**. Retina.

[13] Colucciello M (2006). Choroidal neovascularization complicating photodynamic therapy for central serous retinopathy. Retina.

[14] Lee PY, Kim KS, Lee WK (2009). Severe choroidal ischemia following photodynamic therapy for pigment epithelial detachment and chronic central serous chorioretinopathy. Jpn J Ophthalmol.

[15] Kim KS, Lee WK, Lee SB (2014). Half-dose photodynamic therapy targeting the leakage point on the fluorescein angiography in acute central serous chorioretinopathy: a pilot study. Am J Ophthalmol.

[16] Chen SN, Hwang JF, Tseng LF, Lin CJ (2008). Subthreshold diode micropulse photocoagulation for the treatment of chronic central serous chorioretinopathy with juxtafoveal leakage. Ophthalmology.

[17] Zhou L, Chong V, Lai K, Huang C, Xu F, Gong Y, Youlidaxi M, Li T, Lu L, Jin C (2019). A pilot prospective study of 577-nm yellow subthreshold micropulse laser treatment with two different power settings for acute central serous chorioretinopathy. Lasers Med Sci.

[18] Behnia M, Khabazkhoob M, Aliakbari S, Abadi AE, Hashemi H, Pourvahidi P (2013). Improvement in visual acuity and contrast sensitivity in patients with central serous chorioretinopathy after macular subthreshold laser therapy. Retina.

[19] Sivaprasad S, Elagouz M, McHugh D, Shona O, Dorin G (2010). Micropulsed diode laser therapy: evolution and clinical applications. Surv Ophthalmol.

[20] Yu AK, Merrill KD, Truong SN, Forward KM, Morse LS, Telander DG (2013). The comparative histologic effects of subthreshold 532- and 810-nm diode micropulse laser on the retina. Invest Ophthalmol Vis Sci.

[21] Zhao M, Zhang F, Chen Y, Dai H, Qu J, Dong C, Kang X, Liu Y, Yang L, Li Y (2015). A 50% vs 30% dose of verteporfin (photodynamic therapy) for acute central serous chorioretinopathy: one-year results of a randomized clinical trial. JAMA ophthalmol.

[22] Ozkaya A, Alkin Z, Ozveren M, Yazici AT, Taskapili M (2016). The time of resolution and the rate of recurrence in acute central serous chorioretinopathy following spontaneous resolution and low-fluence photodynamic therapy: a case-control study. Eye.

[23] Koss MJ, Beger I, Koch FH (2012). Subthreshold diode laser micropulse photocoagulation versus intravitreal injections of bevacizumab in the treatment of central serous chorioretinopathy. Eye.

[24] van Rijssen TJ, van Dijk EHC, Scholz P, Breukink MB, Blanco-Garavito R, Souied EH, Keunen JEE, MacLaren RE, Querques G, Fauser S (2019). Focal and Diffuse Chronic Central Serous Chorioretinopathy Treated With Half-Dose Photodynamic Therapy or Subthreshold Micropulse Laser: PLACE Trial Report No. 3. American journal of ophthalmology.

[25] Kim JY, Park HS, Kim SY. Short-term efficacy of subthreshold micropulse yellow laser (577-nm) photocoagulation for chronic central serous chorioretinopathy = Albrecht von Graefes Archiv fur klinische und experimentelle Ophthalmologie. Graefe’s Arch Clin Exp Ophthalmol. 2015;253(12):2129–35. 10.1007/s00417-015-2965-7 25717024

[26] Arora S, Sridharan P, Arora T, Chhabra M, Ghosh B (2019). Subthreshold diode micropulse laser versus observation in acute central serous chorioretinopathy. Clin Exp Optom.

[27] Fujita K, Yuzawa M, Mori R (2011). Retinal sensitivity after photodynamic therapy with half-dose verteporfin for chronic central serous chorioretinopathy: short-term results. Retina.

